# Chicago Medical Society—Regular Meeting, June 6, 1862

**Published:** 1862-06

**Authors:** 


### CHICAGO MEDICAL SOCIETY.—REGULAR MEETING, June 6, 1862.

After the reading and approval of the minutes of the last meeting, Dr. G. Paoli reported a case of scarlatina.

Dr. Fisher related two interesting cases of gun-shot wounds. The first, was a soldier, who in the battle of Shiloh or Pittsburg Landing, received a wound from a Minnie ball, that entered the deltoid muscle, thoroughly shattered both the neck and head of the humerus, grazed the edge of the glenoid cavity, and passed under the scapula, lodging deep in the muscles between the scapula and spine. Several days had elapsed after the wound was received before the patient came under Dr. Fisher's care. An operation was commenced with the hope that exsection of the head of the humerus would be sufficient, but the bone was found so extensively shattered or comminuted that the operation was converted into an amputation at the shoulder-joint. The humerus showing the direction of the ball and the extent of the injury to that bone, was exhibited to the Society. Owing to the feebleness of the patient, and the absence of any indications of serious irritation at the point where the ball was lodged, no attempt was then made to extract it. Under the influence of rest, tonics, stimulants, and good diet the patient soon began to improve, and continued doing well until the wounds connected with the amputation at the shoulder had nearly healed up, and he had been taken home to his friends.

The surgeon, under whose care he then came, made an incision behind the posterior edge of the scapula and extracted the ball. Air was found to escape freely from the wound thus made; great difficulty of breathing soon followed, and the patient died.

On making a post mortem examination, it was found that the ball, in passing under the scapula, had fractured three ribs, cutting one entirely through, and wounding the surface of the lung. It was through this wound that the air escaped after the extraction of the ball, and to which the fatal result was attributable. It is remarkable that such a wound should exist several weeks, without either becoming closed up by adhesive inflammation, or allowing the air to escape both into the pleura and the external tissues sufficiently to render them emphysematous. The second case related by Dr. FISHER, was that of a soldier who, in the same battle, received a ball through the soft parts of the thigh, in such direction as to sever the femoral artery. Secondary hemorrhage occurred, which was not controlled until both ends of the artery were tied. The incisions necessary to effect the ligation of the artery also enabled him to extract the ball. The patient subsequently recovered.

PUERPERAL, PYEMIA, AND FEVER.

Dr. J. McALLISTER related two cases of what he styles puerperal pyemia. In both cases the symptoms were nearly the same. The patients appeared to be doing perfectly well until about the fourth day after confinement; when the attack was ushered in by certain striking nervous symptoms. There was no chill or abdominal pain or tenderness, but the countenance rather suddenly asssumed an expression of anxiety; the mind became confused; and the patient felt as if she was being lifted from the bed and floating in the atmosphere. The acceleration of pulse and febrile heat were very moderate.

In the first case, these symptoms continued with but little change for four days, when there seemed to be a decided improvement. On the fifth day, however, a very disagreeable odor was emitted from the body of the patient, and the vaginal discharge was offensive. On the sixth day, a severe chill occurred, followed by an extremely rapid and feeble pulse, and death the following morning. The second case, presented the same train of symptoms, but reached a fatal termination on the fifth day. In neither case was there any abdominal pain, tenderness, or tympanitis. Dr. ROSS stated that two cases of ordinaay well marked puerperal peritonitis had occurred in his practice recently; one of which terminated fatally on the fifth day after the attack, and the other recovered.

CANCER OF THE STOMACH.

Dr. Ross, also presented an interesting pathological specimen, consisting of the stomach taken from a patient in the hospital, who had died after several years of illness. His chief symptoms during life were vomiting after meals and constipation, with a slow but persistent loss of flesh and strength. The lower and posterior surface of the stomach was closely adherent to the transverse part of the colon. Internally it showed an extensive scirrhus thickening and growth, extending from near the cardiac orifice over the posterior internal surface to the larger curvature. But it did not involve either the cardiac or pyloric orifices.

Dr. Ernst Schmidt, called the attention of the Society to the fact, that in Germany, cancer of the stomach is very much more frequent in those districts where the poorer classes make free use of a poor quality of wine—the refuse of the vintage, than in those where the same classes use lager beer as the common beverage. This, if true, would seem to show that the irritation of the stomach caused by the frequent contact of sour and inferior wine, was a positive *cause* of scirrhus.

TETANUS.

Dr. Schmidt, also related two cases recently occurring in his practice, of spontaneous or idiopathic tetanus. In one of them a hot bath, rendered strongly alkaline by liquor potassa, afforded very marked, though temporary relief. Both cases terminated fatally, but no post mortem examination was allowed in either.

SANITARY REPORT.

Dr. N. S. Davis, presented a report on the Sanitary regulations of the city, with suggestions for their improvement. This report may be found in full, with only slight alterations of phraseology, in the editorial department of this No. of the Examiner.

PERNICIOUS FEVER.

This being the subject for discussion was taken up and briefly discussed by Drs. DAVIS, SCHMIDT, PAOLI, PETERSON, FISHER, C. G. SMITH, WICKERSHAM, and MCALLISTER. The latter made some interesting statements in relation to the prevalence of this form of disease among the prisoners in Camp Douglas, and its connection with pneumonia.

But as a discussion of the subject will be resumed at the next meeting of the Society, we will defer our report until that time.

